# Expression Variation of *CPT1A* Induces Lipid Reconstruction in Goat Intramuscular Precursor Adipocytes

**DOI:** 10.3390/ijms241713415

**Published:** 2023-08-29

**Authors:** Yinmei Tang, Wenyang Zhang, Yinggui Wang, Haiyang Li, Changhui Zhang, Yong Wang, Yaqiu Lin, Hengbo Shi, Hua Xiang, Lian Huang, Jiangjiang Zhu

**Affiliations:** 1 Qinghai-Tibetan Plateau Animal Genetic Resource Reservation and Utilization Key Laboratory of Sichuan Province, Southwest Minzu University, Chengdu 610225, China; yinmeitang2000@126.com (Y.T.); yingguiwang326@163.com (Y.W.); haiyang6715@163.com (H.L.); zhangchanghui0074@163.com (C.Z.); wangyong010101@hotmail.com (Y.W.); linyq1999@163.com (Y.L.); xianghua2008411@163.com (H.X.); 80300227@swun.edu.cn (L.H.); 2 Key Laboratory of Qinghai-Tibetan Plateau Animal Genetic Resource Reservation and Utilization, Southwest Minzu University, Ministry of Education, Chengdu 610041, China; zhangwenyang6588@163.com; 3 College of Animal Science, Zhejiang University, Hangzhou 310058, China; hengboshi@yahoo.com

**Keywords:** IMF, intramuscular adipocyte, *CPT1A*, RNA-seq, MAPK signaling pathway

## Abstract

Intramuscular fat (IMF) deposition is one of the most important factors affecting meat quality and is closely associated with the expression of carnitine palmitoyl transferase 1A (*CPT1A*) which facilitates the transfer of long-chain fatty acids (LCFAs) into the mitochondria. However, the role of how *CPT1A* regulates the IMF formation remains unclear. Herein, we established the temporal expression profile of *CPT1A* during the differentiation of goat intramuscular precursor adipocytes. Functionally, the knockdown of *CPT1A* by siRNA treatment significantly increased the mRNA expression of adipogenic genes and promoted lipid deposition in goat intramuscular precursor adipocytes. Meanwhile, a *CPT1A* deficiency inhibited cell proliferation and promoted cell apoptosis significantly. *CPT1A* was then supported by the overexpression of *CPT1A* which significantly suppressed the cellular triglyceride deposition and promoted cell proliferation although the cell apoptosis also was increased. For RNA sequencing, a total of 167 differential expression genes (DEGs), including 125 upregulated DEGs and 42 downregulated DEGs, were observed after the RNA silencing of *CPT1A* compared to the control, and were predicted to enrich in the focal adhesion pathway, cell cycle, apoptosis and the MAPK signaling pathway by KEGG analysis. Specifically, blocking the MAPK signaling pathway by a specific inhibitor (PD169316) rescued the promotion of cell proliferation in *CPT1A* overexpression adipocytes. In conclusion, the expression variation of *CPT1A* may reconstruct the lipid distribution between cellular triglyceride deposition and cell proliferation in goat intramuscular precursor adipocyte. Furthermore, we demonstrate that CPT1A promotes the proliferation of goat adipocytes through the MAPK signaling pathway. This work widened the genetic regulator networks of IMF formation and delivered theoretical support for improving meat quality from the aspect of IMF deposition.

## 1. Introduction

Goat meat has the advantage of being high in protein and low in cholesterol. These features can decrease the risk of heart-related diseases caused by a high lipid intake [1]. Moreover, more and more people are choosing goat meat as a more reliable and safer option due to the negative impact of the African swine fever and avian flu viruses [2,3]. Goat meat has become a superior food for the national market and is widely consumed on a global scale with not only an increasing quantity, but also an improving quality demand [4,5]. Considering the importance of intramuscular fat (IMF) [6] for meat quality promotion, it is believed to be important to elucidate the molecular mechanism underlying IMF deposition and fatty acid metabolism, so as to improve the quality of goat meat.

Carnitine palmitoyltransferase I (CPT1) is located in the inner side of the outer mitochondrial membrane. It catalyzes the reversible transfer of acyl groups from specific chain-length acyl-CoA esters to carnitine to form acylcarnitine esters [7]. Carnitine palmitoyltransferase I hepatic isoform (CPT1A), the most widely distributed isoform of CPT1, is an enzyme that plays a decisive role in fatty acid oxidation by loading lipid acyl groups onto carnitine to transfer long-chain fatty acids into mitochondria [8]. *CPT1A* is expressed in liver, pancreas, kidney, skeletal muscle, brain, blood and embryonic tissues [9]. As well as being related to various cancers [10,11,12,13,14,15,16,17] and rat liver [18], *CPT1A* is significantly positively correlated with the IMF deposition in pig longissimus dorsi [19]. Our previous study also supported the truth that a higher expression of *CPT1A* is terminally significantly correlated (*p* = 0.002, *r* = 0.934) with the longissimus dorsi IMF content in goats [20]. However, in chicken breast muscle, the lower expression of *CPT1A* was matched with a higher IMF content in Wenchang Chickens compared to White Recessive Rock chickens with a lower IMF content, which was then validated by the functional determination in quail muscle myoblasts (QM-7 cells) [21]. Considering the differences among different species, it is meaningful to explore the molecular mechanisms by which high CPT1A expression promotes IMF deposition in goats.

We also realized that an increase in the number of adipocytes is another important factor influencing IMF formation. The expression of CPT1A significantly inhibited cell proliferation in renal cancer cells [22] and MDA-MB231 breast cancer cells [23], but activated cell proliferation in prostate cancer cells [24] and in V600E melanoma [25]. However, studies on the role of CPT1A in livestock are scarce. The regulatory role of CPT1A for cell proliferation during IMF formation in goats remains unclear. Taking both cellular lipid deposition and cell proliferation into consideration may be beneficial for comprehensively understanding the role of *CPT1A* in regulating IMF formation.

In the present study, we explored the function of *CPT1A* in intramuscular adipocytes by RNA silencing and overexpression. Moreover, the RNA-seq was carried out to screen the differential transcripts affected by *CPT1A* function loss. At last, these data illuminated the possibility that *CPT1A* may be involved in IMF regulation by inhibiting cellular lipid deposition, but promoting cell proliferation and, as a result, increasing the IMF content in goats.

## 2. Results

### 2.1. Knockdown of CPT1A Promotes Lipid Deposition in Goat Intramuscular Precursor Adipocytes

**Figure 1:**
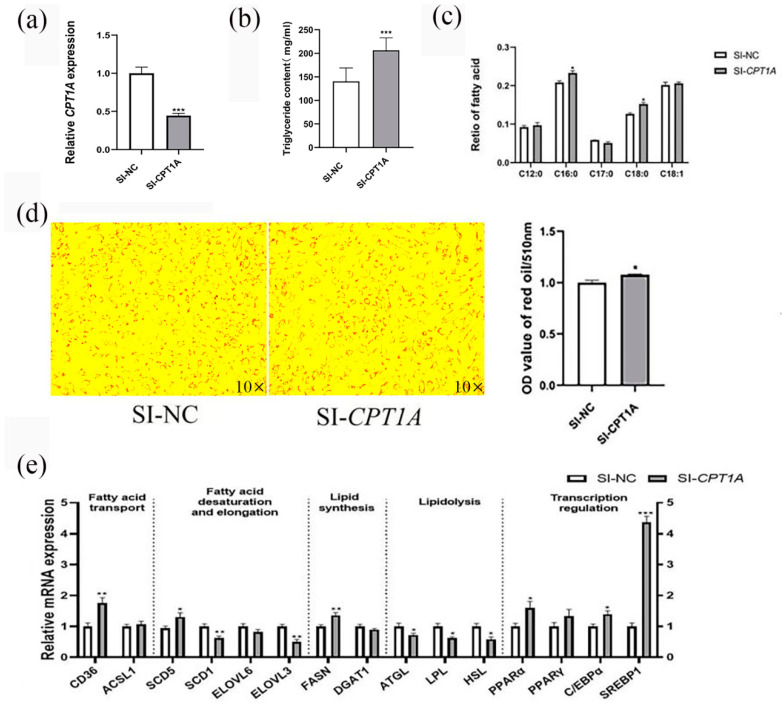
Knockdown of *CPT1A* promotes lipid deposition in goat intramuscular precursor adipocytes. (**a**) Knockdown efficiency detection. With UXT as the internal reference gene and the negative control as reference. (**b**) Triglyceride content in *CPT1A* knockdown cells. (**c**) Effect of *CPT1A* knockdown on fatty acid profiles in goat adipocytes. (**d**) Knockdown of *CPT1A* (SI-*CPT1A*) after oil red O staining, OD measurement by oil red O staining extraction. (**e**) Effect of *CPT1A* knockdown on the expression levels of genes related to lipid metabolism. Data are presented as mean ± SEM for three independent experiments. * *p* < 0.05, ** *p* < 0.01, *** *p* < 0.0001.

**Figure 2:**
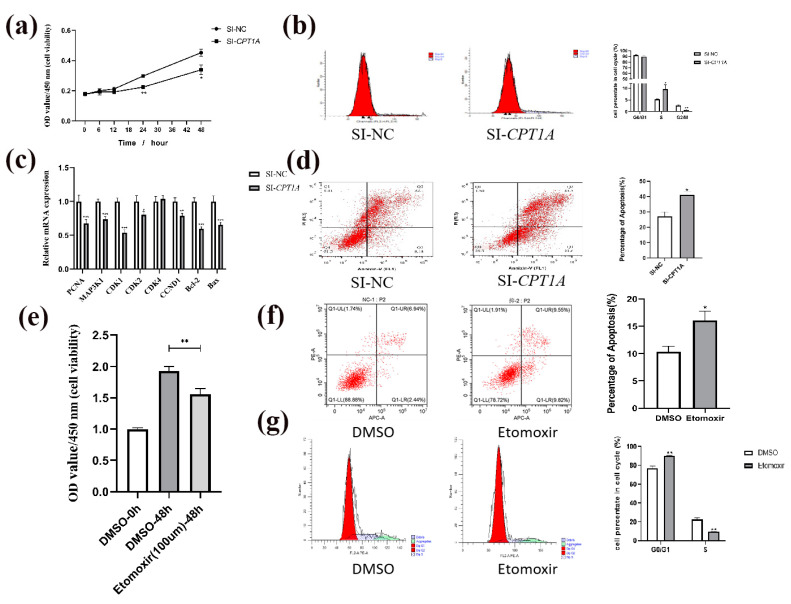
Effect of *CPT1A* reduction on the proliferation, cycle and apoptosis of goat adipocytes. (**a**) Knockdown of *CPT1A* inhibits the cell proliferation of goat adipocytes, OD value of 450 nm characterizes cell viability. (**b**) Effect of *CPT1A* deficiency on the cycle in goat adipocytes. (**c**) Effect of knockdown of *CPT1A* on the expression levels of genes related to cell proliferation, cell cycle and apoptosis. (**d**) Effect of *CPT1A* deficiency on the apoptosis in goat adipocytes. (**e**) Determination of Etomoxir concentration in goat adipocytes. (**f**) Effect on apoptosis after Etomoxir treatment in goat adipocytes. (**g**) Effect on cell cycle after Etomoxir treatment in goat adipocytes. Data are presented as mean ± SEM for three independent experiments. * *p* < 0.05, ** *p* < 0.01, *** *p* < 0.0001.

To elucidate the role of *CPT1A* in regulating intramuscular lipid accumulation in goats, SI-*CPT1A* was transfected into goat intramuscular preadipocytes to perform loss of function. Lipid droplets, the triglyceride (TAG) content, the composition of fatty acids and the expression of the lipid metabolism-related gene after deleting *CPT1A* were estimated. The results showed that the mRNA expression of *CPT1A* was significantly reduced by 70.30% after treatment with SI-*CPT1A* compared to the control group (*p* < 0.001, Figure 1a). The relative content of TAG was increased after *CPT1A* knockdown (*p* < 0.001, Figure 1b). Likewise, the lipid droplets content was significantly increased after *CPT1A* knockdown by Oil Red O staining (*p* < 0.05, Figure 1d) and Bodipy staining (*p* < 0.001, Supplementary Materials Figure S1). In addition, the total fatty acids were detected in the goat adipocytes. Among them, the percentage of palmitic acid (C16:0) and stearic (C18:0) increased significantly after SI-*CPT1A* transfection (*p* < 0.05, Figure 2c). The percentage of C12:0, C17:0 and C18:1 did not significantly change (Figure 1c). To investigate the expression changes in the lipid metabolism-related genes caused by *CPT1A* knockdown, we assessed the expression of these genes in *CPT1A* knockdown cells (Figure 1e). The knockdown of *CPT1A* increased the expression of the fatty acid transport gene *CD36* (*p* < 0.01) and the fatty acid desaturation gene *SCD5* (*p* < 0.05), while decreasing the expression of the fatty acid desaturation gene *SCD1* (*p* < 0.01). The expression of the fatty acid elongation gene *ELOVL3* was decreased in the knockdown cells (*p* < 0.01); the key de novo fatty acid synthesis gene *FASN* (*p* < 0.01) expression was significantly increased after *CPT1A* knockdown. The key genes related to lipolysis, *ATGL*, *LPL*, *HSL* (*p* < 0.05), were significantly decreased after *CPT1A* knockdown. Compared with SI-NC, the knockdown of *CPT1A* increased the transcription of transcription regulators PPARα, *C/EBPα (p* < 0.05) and *SREBP1* (*p* < 0.001). The expression of *ACSL1*, *DGAT1*, *ELOVL6* and *PPARγ (p* > 0.05) were not significantly changed.

### 2.2. Reduction of CPT1A Inhibits Cell Proliferation in Goat Intramuscular Precursor Adipocytes

With the purpose of exploring the function of *CPT1A* in goat adipocyte proliferation, a CCK-8 assay and flow cytometry were used to detect the proliferation of goat adipocytes. The results showed a significant decrease in cell viability (OD value of 450 nm) in the knockdown *CPT1A* group compared to the control group (24 h and 48 h, Figure 2a). The mRNA expression levels of the proliferation-related genes were examined and the results showed that the knockdown of *CPT1A* significantly reduced the mRNA level of *MAP3K1* (*p* < 0.0001), *PCNA* (*p* < 0.0001), *CDK1* (*p* < 0.0001), *CDK2* (*p* < 0.05) and *CCND1* (*p* < 0.01), but did not affect the mRNA level of *CDK4* (Figure 2c).

A study demonstrates that the intake of conjugated linoleic acid reduces adipose tissue by apoptosis, which shows that apoptosis and fat deposition are inextricably linked [26]. As a result, the knockdown of *CPT1A* promoted apoptosis in goat adipocytes (*p* < 0.05, Figure 2d). In order to protect cells from interfering RNA toxicity, we validated the results by treating the cells with a specific *CPT1A* inhibitor (Etomoxir), using a concentration of 100 μm. The results showed that the 100 μm Etomoxir-treated group showed significantly lower cell viability than the control group at 48 h (*p* < 0.01, Figure 2e). Etomoxir treatment promoted apoptosis in goat adipocytes (*p* < 0.05, Figure 2f). The mRNA expression levels of the apoptosis-related genes were examined, and the results showed that the overexpression of *CPT1A* significantly reduced the mRNA level of *Bcl-2* and *Bax* (*p* < 0.001, Figure 2c).

Recent studies have shown that eicosapentaenoic acid induces meibocyte differentiation through *PPARγ* activation that is characterized by the cell cycle exit, de novo and transported lipid accumulation [27]. The proportion of S-phase increases and the proportion of G2/M-phase decreases after interference with *CPT1A* (Figure 2b). The proportion of G0/G1 increases and the proportion of S-phase decreases after Etomoxir treatment (Figure 2g).

### 2.3. Overexpression of CPT1A Inhibits Lipid Deposition in Goat Intramuscular Precursor Adipocytes

**Figure 3:**
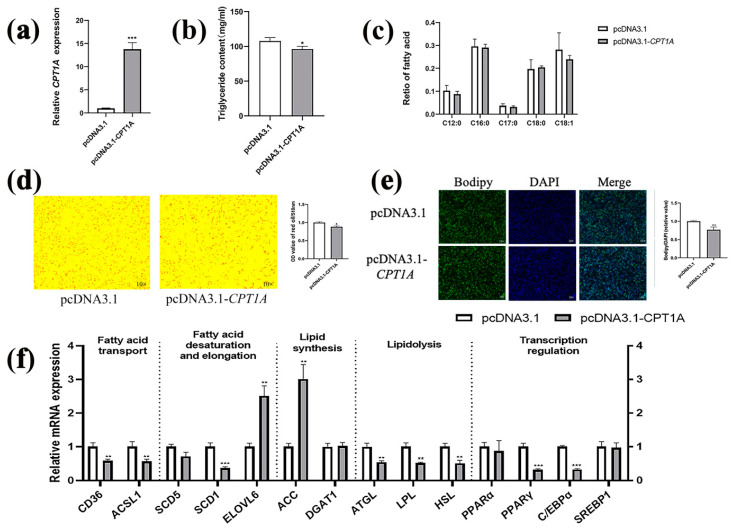
Overexpression of *CPT1A* inhibits lipid deposition in goat intramuscular precursor adipocytes. (**a**) Overexpression efficiency detection. With *UXT* as the internal reference gene and the pcDNA3.1 as reference. (**b**) Triglyceride content in *CPT1A* overexpression cells. (**c**) Effect of *CPT1A* overexpression on fatty acid profiles in goat adipocytes intramuscular precursor cells. (**d**) Overexpression of *CPT1A* (pcDNA3.1-*CPT1A*) after oil red O staining, OD measurement by oil red O staining extraction. (**e**) Overexpression of *CPT1A* after Bodipy staining, quantification of lipid droplet content. (**f**) Effect of *CPT1A* overexpression on the expression levels of genes related to lipid metabolism. Data are presented as mean ± SEM for three independent experiments. * *p* < 0.05, ** *p* < 0.01, *** *p* < 0.0001.

Our results provide evidence that *CPT1A* knockdown promotes intramuscular preadipocytes lipid deposition. Here, we constructed pcDNA3.1-*CPT1A* to overexpress *CPT1A* and transfected goat intramuscular preadipocytes to further elucidate the role of *CPT1A* through gain of function. The results showed that the *CPT1A* expression of the pcDNA3.1-*CPT1A* group was upregulated 13-fold using the transfected pcDNA3.1 group as a negative control group (*p* < 0.001, Figure 3a). Correspondingly, the relative content of TAG was decreased after *CPT1A* overexpression (*p* < 0.05, Figure 3b). Likewise, the lipid droplets content was significantly decreased after *CPT1A* overexpression by Oil Red O staining (*p* < 0.05, Figure 3d) and Bodipy staining (*p* < 0.001, Figure 3e). However, the percentage of C12:0, C16:0, C17:0, C18:0 and C18:1 did not significantly change (Figure 3c). To investigate the expression changes of Lipid metabolism-related genes caused by *CPT1A* overexpression, we assessed the expression of these genes in *CPT1A* overexpression cells (Figure 3f). The overexpression of *CPT1A* decreased *CD36* (*p* < 0.01), *ACSL1* (*p* < 0.01), *SCD1* (*p* < 0.001), *ATGL* (*p* < 0.01), *LPL* (*p* < 0.01), *HSL* (*p* < 0.01), *PPARγ* (*p* < 0.001) and *C/EBPα* (*p* < 0.001). The *ELOVL6* and *ACC* genes’ expressions were significantly increased after *CPT1A* overexpression. The expression of *SCD5*, *DGAT1*, *PPARα* and *SREBP1* (*p* > 0.05) were not significant changed compared to the pcDNA3.1 group.

### 2.4. Overexpression of CPT1A Promotes Cell Proliferation in Goat Intramuscular Precursor Adipocytes

**Figure 4:**
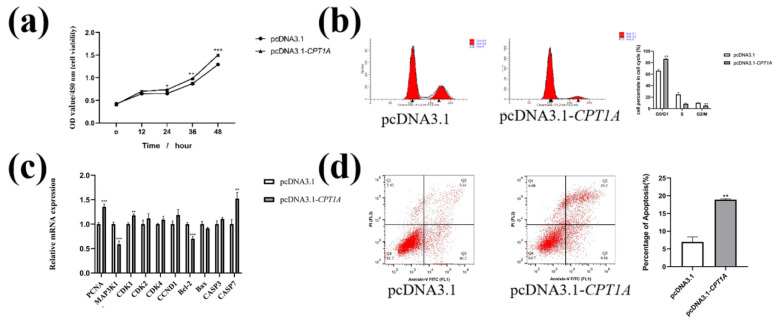
Effect of overexpression of *CPT1A* on the proliferation, cycle and apoptosis of goat adipocytes. (**a**) Overexpression of *CPT1A* promotes cell proliferation in goat intramuscular precursor adipocytes. (**b**) Effect of *CPT1A* overexpression on the cycle in goat adipocytes. (**c**) Effect of overexpression of *CPT1A* on the expression levels of genes related to cell proliferation, cell cycle and apoptosis. (**d**) Effect of *CPT1A* promotion on apoptosis in goat adipocytes. Data are presented as mean ± SEM for three independent experiments. * *p* < 0.05, ** *p* < 0.01, *** *p* < 0.0001.

Based on the results that the knockdown of *CPT1A* inhibits cell proliferation, in order to verify whether the overexpression *CPT1A* could promote cell proliferation, we used CCK-8 to test whether cell viability could be enhanced after the overexpression of *CPT1A*. The results showed that the pcDNA3.1*-CPT1A* group showed a significant increase in cell viability at 24 h (*p* < 0.05), 36h (*p* < 0.01) and 48h (*p* < 0.0001*)* compared to the pcDNA3.1 group. Further proliferation-related gene expression analysis showed that *CPT1A* overexpression significantly increased the mRNA levels of *PCNA* (*p* < 0.0001), *CDK1* (*p* < 0.01) and *CDK4* (*p* < 0.05), but significantly decreased the mRNA levels of *MAP3K1* (*p* < 0.0001). The mRNA level of *CCND1* and *CDK2* did not significantly change (Figure 4c). As like the knockdown of *CPT1A*, the overexpression of *CPT1A* promoted apoptosis in goat adipocytes (*p* < 0.05, Figure 4d). Further apoptosis-related gene expression analysis showed that the overexpression of *CPT1A* significantly reduced the mRNA levels of *Bcl-2* (*p* < 0.0001) and significantly increased the mRNA levels of *CASP7* (*p* < 0.001, Figure 4c). The mRNA levels of *Bax* and *CASP3* were not changed. The results showed that the cell was arrested in G0/GI after the overexpression of *CPT1A* (*p* < 0.05, Figure 4d) and the G2/M phase significantly reduced.

### 2.5. Silencing of CPT1A Change Cellular Gene Expression Profile in Goat Preadipocytes

**Figure 5:**
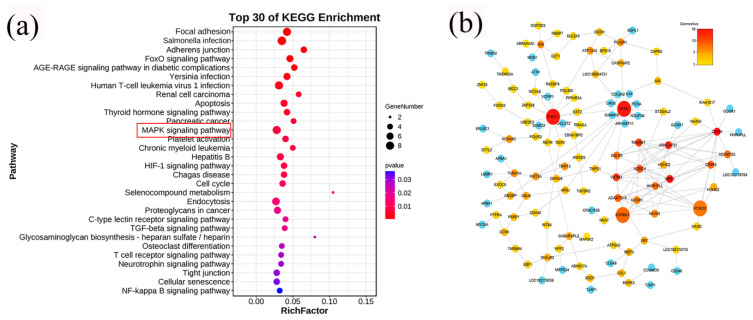
DEGs analysis after *CPT1A* knockdown. (**a**) The top 30 enriched pathways of DEGs. Pathways in red boxes indicate pathways that were subsequently studied. (**b**) Visual network diagram for correlation analysis of differentially expressed gene expression uses the software of Cytoscape 3.9.1 for graphing, gradient colors indicate the strength of the DEGs correlation (1–19).

To further investigate the mechanism of action of *CPT1A* in inhibiting lipid deposition, we sequenced and analyzed the transcriptome of total RNA from the goat adipocytes knockdown of *CPT1A* and negative controls. The volcano map showed (Supplementary Materials Figure S2 and Table S1) that a total of 167 differentially expressed genes (DEGs) were screened (*p* < 0.05, Fold change > 1), of which 125 were upregulated and 42 were downregulated. The heat map showed (Supplementary Materials Figure S2 and Table S2) that with large differences between groups, the expression patterns were similar between samples within groups, indicating small differences between samples. In the GO database, DEGs were divided into three catalogs (Supplementary Materials Figure S3 and Table S4): molecular function (MF,129), cellular component (CC,141) and biological process (BP,135). The top 30 of the KEGG pathways that were significantly enriched are shown in the figure, such as Focal adhesion, the MAPK signaling pathway, the HIF-1 signaling pathway and the FOXO signaling pathway, and others (Figure 5a, Table S3). Of them, Focal adhesion had the top enrichment, with eight enriched in this pathway. In addition, we used correlation screens between DEGs to analyze genes that may play a key role in the regulation of lipid deposition (Figure 5b, Table S5). The results showed that *PCBD2*, *FNDC1*, *AP2S1* and *SORBS1* genes may be the key genes.

### 2.6. CPT1A Dependence Promotes Proliferation by the MAPK Signaling Pathway

**Figure 6:**
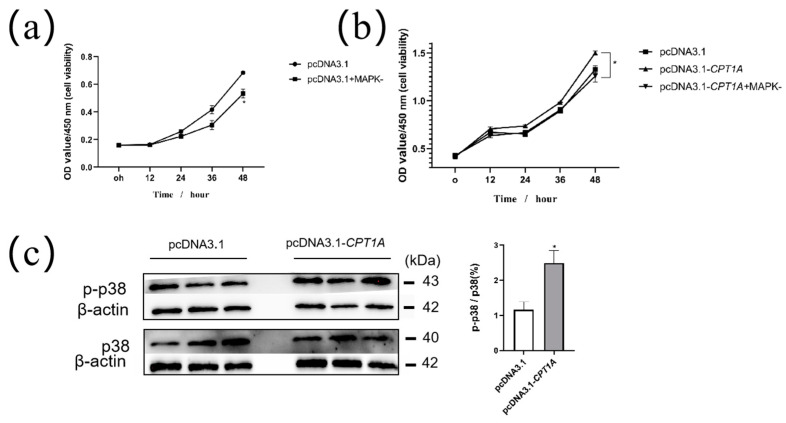
Overexpression of *CPT1A* promotes intramuscular adipocytes proliferation through MAPK signaling pathway. (**a**) MAPK-(PD169316) inhibits proliferation in goat adipocytes. (**b**) The promotion of cell proliferation by overexpression of *CPT1A* was partially counteracted by MAPK-. (**c**) Detection of p-p38 and p38 protein levels changes after overexpression of *CPT1A* using Western Blot. * *p* < 0.05.

RNA-seq analysis showed that the MAPK signaling pathway was enriched in the KEGG enrichment analysis of DEGs. This suggests that the promotion effect of *CPT1A* on cell proliferation may be mediated by the MAPK pathway. To explore it, we enhanced the expression of *CPT1A* followed by the treatment of MAPK pathway inhibitors (PD169316) to observe whether the inhibitor could counteract the promotion of cell proliferation by the overexpression of *CPT1A*. First, we demonstrated the inhibitory effect of the MAPK inhibitor (PD169316) on goat adipocyte proliferation (Figure 6a). The results showed that goat adipocyte activity was significantly inhibited in the pcDNA3.1-*CPT1A* post-addition PD169316 group compared to the pcDNA3.1-*CPT1A* group (Figure 6b). The results of detecting total P38-MAPK protein and phospho-p38-MAPK(p-p38/p38) protein levels showed that the p-p38/p38(%) in the pcDNA3.1-*CPT1A* group was significantly higher than that in the pcDNA3.l group (Figure 6c). These results imply that *CPT1A* is regulated by the MAPK signaling pathway and thus affects goat adipocyte proliferation.

## 3. Discussion

*CPT1A* is closely linked to fat deposition by effecting lipid metabolism and cell proliferation. However, the mechanisms underlying the role of *CPT1A* in regulating lipid metabolism and cell proliferation remain elusive. Our results demonstrate that *CPT1A* negatively regulates lipid deposition while positively regulating cell proliferation in goat adipocytes via the MAPK signaling pathway. Moreover, RNA-seq data imply that the screened DEGs are expected to further complement the network of molecular mechanisms of IMF deposition.

At present, research on *CPT1A* primarily focuses on disease treatment. However, it has also been reported to have a role in animal lipid deposition, with studies conducted on pigs [19], mice [21] and chickens [28]. On the basis of the cloned goat *CPT1A* gene sequence, the present study investigated the regulatory role of *CPT1A* on IMF deposition using RNA silencing and gene overexpression. The results indicated that the overexpression of *CPT1A* significantly reduced the triglyceride content and lipid accumulation in goat adipocytes. Moreover, consistently overexpressing *CPT1A* attenuated lipid accumulation in the clear renal cancer cells [29]. Additionally, the increased *CPT1A* expression significantly reduced hepatic triglyceride levels in obese mice and lean rats [28]. Fatty acids are critical for lipid deposition and it is well known that C16:0 is produced via FASN enzymes inducing fatty acid de novo synthesis [30]. The increased content of C16:0 may be caused by the upregulation *FASN* mRNA in the knockdown of *CPT1A* cells. *SCD5* preferentially desaturates two saturated fatty acids, C16:0 and C18:0, into monounsaturated fatty acids, palmitoleic (C16:1) and oleic (C18:1) [31]. We speculate that the increase in C16:0 and C18:0 caused an upregulation of *SCD5* gene expression in the knockdown of *CPT1A* cells. The overexpression of *CPT1A* significantly reduced the expression levels of the *CD36*, *SCD1*, *PPARγ* and *C/EBPα* genes, thus presumably reducing lipid deposition by inactivating transcription factor activity and limiting fatty acid intake and absorption. The knockdown of *CPT1A* significantly increased the expression of *CD36* and *SCD5* genes and significantly decreased the expression of *LPL*, *HSL* and *SCD1* genes. Thus, we hypothesized that the promotion of lipid deposition after knocking down *CPT1A* might be achieved by increasing lipid synthesis and decreasing lipid catabolic pathways.

The contents of IMF are mainly determined by the number and size of intramuscular adipocytes, of which the number of intramuscular adipocytes is especially important [32], as well as apoptosis [33]. What is more, the amount, composition and localization of the lipid repertoire are dynamic in dividing cells and changes in the cell cycle affect lipid metabolism [34]. In the present study, the function deficiency of *CPT1A* by RNA silencing inhibited cell proliferation in goat preadipocytes, consistent with the observation in human leukemia cells that the pharmacologic inhibition of fatty acid oxidation with etomoxir or ranolazine inhibited proliferation and sensitized human leukemia cells to apoptosis induction [35]. Apoptosis, another factor that affects cell numbers, deserves to be detected. Our results showed that the knockdown of *CPT1A* and Etomoxir treatment promoted apoptosis in goat adipocytes. Consistently, it has been demonstrated that the knockdown of *CPT1A* reduces fatty acid oxidation and leads to apoptosis in colorectal cancer cells [17]. Another study in line with this proved that the genetic deletion of *CPT1A* augmented apoptosis in endothelial cells under hyperoxigenated conditions [36]. Interestingly, in the present study, the upregulated expression of *CASP7* (a pro-apoptotic regulator) and downregulated expression of *Bcl-2* (an anti-apoptotic regulator) by *CPT1A* overexpression implied their involvement in apoptosis regulation. However, the mechanism by which the overexpression of *CPT1A* promotes apoptosis should be further explored. Our results showed a significant increase in the S phase and a significant decrease in G2/M after CPT1A knockdown, and that the cell cycle was arrested in G0/GI after CPT1A overexpression. However, the inactivation of *CPT1A* induced cell cycle arrest at G0/G1, suggesting that ovarian cancer cells depend on or are addicted to *CPT1A*-mediated FAO for cell cycle progression [13]. Another study showed that targeting *CPT1A*-mediated FAO hinders the cell cycle G1/S transition [37]. Considering the toxic effects of interfering RNA on cells, we used a *CPT1A* inhibitor (Etomoxir) to improve our results, the results were consistent with previous studies which showed that *CPT1A* inactivation resulted in cell cycle arrest at the G0/G1 phase. Although the cell type differences obviously should be taken into account, the exact mechanism underlying cell proliferation still needs to be focused more on goats. In addition, the effect of *CPT1A* on apoptosis may be one of the factors contributing to cycle changes, whereas the effect of *CPT1A* on cell proliferation may be an important factor regulating IMF deposition in goat adipocytes.

This study further analyzed the potential mechanism of *CPT1A* regulating IMF deposition using RNA-seq. GO enrichment analysis showed that DEGs were enriched by the cellular oxidative stress process, lipid granules and lipid acyl-CoA catabolic processes, indicating that *CPT1A* is closely linked to lipid metabolism. The KEGG enrichment analysis of DEGs showed the significant enrichment of the FOXO signaling pathway, Focal adhesion pathway, MAPK signaling pathway, Apoptosis, Cell Cycle et al. FOXO, a subfamily of the fork-head transcription factor family, and the FOXO gene are capable of regulating the process of cell differentiation [38]. In different cancers, FOXO interacts with the PI3K/AKT pathway, which is closely related to lipid metabolism [39]. In addition, FOXO can be regulated by insulin in cancer cells and thus participate in lipid metabolism [40]. This shows that the FOXO signaling pathway is essential for lipid metabolism, but gaps remain in its role in regulating lipid deposition in goats, and its exploration is necessary for future research. According to Tholen, the *PCBD2* gene is abundant in adipose tissue and is associated with type II diabetes. In addition, *PCBD2* can interact with HNF1β to influence cell growth and individual development by exerting enzymatic and transcriptional activity [41]. In a proteomic study, FNDC1 protein was identified to be involved in lipid metabolism and transport in rats [42]. In a genome-wide DNA methylation analysis of leukocytes from high LDL-cholesterol obese and low LDL-cholesterol obese individuals, the promoter of *AP2S1* was hypermethylated, as well as *CPT1A* [43]. This suggests that AP2S1 is involved in the lipoprotein metabolism and *AP2S1* is linked with *CPT1A*. *SORBS1* has been shown to be involved in the lipid metabolism [44,45] and other studies have proven that *SORBS1* is associated with obesity and insulin signaling [46]. In addition, it has been shown that *SORBS1* is a candidate gene that affects fatty acid traits in milk [47], thus we presume that it may affect the fatty acid composition of goat meat in IMF deposition. Further analysis of these potential genes regulating intramuscular fat deposition in goats will likely partially complement the molecular mechanisms regulating intramuscular fat deposition in goats.

It has been shown recently that the p38 MAPK signaling pathway promotes cell proliferation [48]. Previous research showed that PD169316 was a specific inhibitor of p38 MAPK by inhibiting the phosphorylation and nuclear translocation of SMAD2 and SMAD3 induced by TGFβ [49], and it has been well studied in human ovarian cancer cells [50] and in equine digital vein endothelial cells [51]. Thus, it was used for determining whether *CPT1A* promotes the proliferation of goat adipocytes by the MAPK signaling pathway. Our results imply that *CPT1A* is regulated by the MAPK signaling pathway and thus affects goat adipocyte proliferation. In addition, evidence has been presented showing that *CPT1A* is regulated as a transcriptional target gene by the p38 MAPK signaling pathway and affects the proliferation of hepatocellular carcinoma cells [52]. Evidence is available to prove that estrogen binding to *GPR30* promotes proliferation via the MEK/ERK and PI3K/AKT signaling pathway in goat mammary epithelial cells [53]. It is reasonable to speculate that the MAPK signaling pathway can influence lipid deposition, and it would be interesting to explore the MAPK signaling pathway’s regulation of lipid metabolism. Moreover, to further elucidate the role of *CPT1A* regulated by MAPK signaling, a future study is needed to explore the relationship between upstream genes capable of regulating the MAPK pathway and the cascade response to *CPT1A*.

## 4. Materials and Methods

### 4.1. Ethics Statement

All experimental exercises were isolated and approved by the Institutional Animal Care and Use Committee, Southwest Minzu University (Chengdu, China). Permit number: S2020-013, revised in June 2004.

### 4.2. Cell Isolation and Culture

The isolation and culture of goat intramuscular precursor cells were reported with reference to previous studies [54]. Briefly, longissimus dorsi tissue were excised from 2-day-old goats and minced. The intramuscular adipocytes were resuspended using Type II collagenase. Enzymatic digestion was terminated by the equal volume of DMEM/F12 (Gibco, Beijing, China) supplemented with 10% fetal bovine serum (FBS) (Gemin, Beijing, China). The suspension was filtered through a 75 µm cell strainer and then centrifuged at 2000 rpm/min for 5 min. After disposing of the red blood cell lysed solution, the suspension was centrifuged at 2000 rpm/min for 5 min again. The preadipocytes were resuspended in DMEM/F12 supplemented with 10% FBS and diluted to a final concentration of 10^6^ cells/mL. The cells were cultured at 37 °C under a humidified atmosphere containing 50 mL·L-1 CO_2_.

### 4.3. Construction of pc DNA3.1-CPT1A Overexpression Vector and siRNA Synthesis

The subcloning primers were designed based on the complete sequence of *CPT1A* CDS (MH345735.1) previously cloned in the laboratory and uploaded to GenBank. PcDNA3.1 (+) plasmid was double cleaved by *EcoRI* and *HindIII* and ligated to the CDS region of the *CPT1A* gene, named pcDNA-*CPT1A*. Then, the recombinant plasmid was identified by enzyme digestion and DNA sequencing (Supplementary Materials Figure S4). The empty PcDNA3.1 (+) plasmid was used as a negative control, named pcDNA3.1. The siRNA was designed and synthesized using the goat *CPT1A* gene as a target by Shanghai GenePharma Co., Ltd., (Shanghai, China) siRNA-*CPT1A* (S:CGCACCACCAAGAUCUGGA-UGUUUA; AS:UAAACAUCCA GAUCUUGGUGGUGCG) and Negative control (SI-NC) was provided by GenePharma (S:UUCUCCGAACGUGUCACGUTT; AS:ACGUGA-CACGUUCGGAGAATT).

### 4.4. Cell Transfection

Transfection was started when the confluence of intramuscular precursor adipocytes in the 6-well plate reached 80%. The transfection procedure is based on the protocol of Lipofectamine™ 3000, where each 6-well was transfected with 1 μg plasmid or 120 μM siRNA. Fluorescent images of the transfection system are provided in the Supplementary Material (Supplementary Materials Figure S5).

### 4.5. Oil Red O (ORO) Staining and Bodipy Staining

To determine lipid droplets, after washing with PBS, cells were fixed with 4% formaldehyde for 30 min. In the oil red O (ORO) staining process, following staining for 30 min with freshly prepared ORO working solution (the mixture of 3 mL Oil Red 5 g/L dissolved in isopropanol and 2 mL of ddH_2_O), the cells were cleaned with PBS. Finally, images were taken under a microscope. ORO quantification was performed by adding 600 µL of isopropanol to the 6-well plate and determining the absorbance value at 510 nm. In the Bodipy staining process, the cells were incubated with Bodipy staining solution (1 μg/mL of BODIPYTM 493/503 (Thermo Fisher Scientific, Waltham, MA, USA, D3922)) and were kept out of the light for 30 min. Subsequently, cells were incubated with a DAPI staining solution (1 μg/mL of DAPI (Solarbio, Beijing, China, C0060) for 10 min in a shaker protected from light. Finally, cells were photographed and stained with Image-Pro Plus 6.0 to measure the fluorescence intensity and cell number.

### 4.6. Triglyceride (TAG) Content Assay

Triglyceride content was quantified using a triglyceride assay kit (Applygen, Beijing, China, E1013). Briefly, adipocytes were treated with cell lysis buffer and the supernatant was collected. The quantification of triglyceride was normalized to the cellular protein concentration using a BCA protein assay kit. Triglyceride absorbance values were measured at 550 nm and BCA absorbance values were measured at 562 nm; the values were calculated from the standard curve and corrected for triglycerides using BCA.

### 4.7. Total RNA Isolation, cDNA Synthesis and q-PCR Analysis

The total RNA was extracted using RNAiso Plus (Takara, Kusatsu, Japan, 9109). The NanoDrop 2000 spectrophotometer (Thermo Fisher, DE, USA) was used to determine the RNA purity and quantity, and the absorbance ratios (260/280 nm) of all RNAs were between 1.8 and 2.0. Then 1 μg of total RNA was reverse transcribed into cDNA using the Reverse Transcription Kit (Vazyme, Nanjing, China, R323-01). Real-Time PCR was carried out with a Bio-Rad CFX96 PCR System using Taq Pro Universal SYBR qPCR Master Mix (Vazyme, Q712-02) and gene-specific primers (Table S6). UXT was used as an internal reference gene and the relative expression was calculated using the 2^−∆∆CT^ method. Comparisons were made by one-way ANOVA in GraphPad prism 8.0 software, and Duncan’s new multiple range tests were used to analyze statistical significance.

### 4.8. Fatty Acid Analysis

Total intracellular fatty acids were extracted from 60 mm culture dishes using 2 mL of sulfuric acid/methanol (2.5:1, *v*/*v*) and collected in an 8 mL glass tube for methyl esterification, as previously reported [55]. Methylated fatty acids were analyzed by gas chromatography (Agilent 7890B, Agilent Technologies Inc., Santa Clara, CA, USA) with HP-88 column (Agilent Technologies Inc., Santa Clara, CA, USA). Supelco 37 Component FAME Mix (CRM47885, Sigma) was used as external standard. Relative proportions of fatty acids in Intramuscular precursor adipocytes were determined as percentages of the total peak area.

### 4.9. RNA Sequencing (RNA-Seq)

The total RNAs from NC and CPT1A knockdown intramuscular adipocytes were performed. The RNA samples were sent to Hangzhou Lc-bio Technologies for transcriptome sequencing. DEsq2 was used to screen differential genes, and the screening condition was *p* < 0.05 and fold change < 1.5. Gene enrichment was analyzed using Gene Ontology (GO) and Kyoto encyclopedia of genes and genomes (KEGG) databases.

### 4.10. Cell Counting Kit-8 (CCK-8) Assay

CCK-8 assay was used for the evaluation of intramuscular adipocytes proliferation. Cells were seeded into a 96-well plate with 4 replicates per treatment and transfected with SI-NC or SI-*CPT1A*, and pc-DNA 3.1 or pc-DNA 3.1-*CPT1A*, respectively. The final concentration (10 μM, Supplementary Materials Figure S6) of PD169316 (p38 MAPK inhibitor, Beyotime, Shanghai, China) was added to wells. After 0, 12, 24, 36 and 48 h treated by p38 MAPK inhibitor, adding 10 μL CCK-8 reagent (AC11L054, Life-iLab, Shanghai, China) per well, the plates were incubated for 0.5 h at 37 °C. At last, the absorbance was measured using an enzyme-labeled instrument at a wavelength of 450 nm.

### 4.11. Flow Cytometry for Cell Cycle Analysis

Cells inoculated into six-well plates were transfected using the same method as for the CCK-8 assay. After 48 h transfected, the cells were digested, collected, then stained with propidium iodide (C0080, solarbio, Beijing, China) for 30 min at room temperature. Finally, cell cycle classification and statistical analysis was carried out by flow cytometry.

### 4.12. Flow Cytometry for Apoptosis Analysis

The ratio of apoptotic cells was detected by using an Annexin V-FITC/PI Apoptosis Detection kit (A211, Vazyme, Nanjing, China) according to the manufacturer’s instructions. Briefly, the cells were treated using the same method as for the cell cycle analysis. The collected cell suspension was added to 100 μL of 1× binding buffer, then 5 μL AnnexinV-FITC and 5 μL PI were added to the cell suspension.

### 4.13. Immunofluorescence

Cells were fixed in 4% paraformaldehyde and washed with PBS. The samples were permeabilized in 0.25% Triton X-100 and incubated with 5% goat serum albumin for 1 h at room temperature, followed by incubation with a diluted solution (1:300) of following antibody: anti-CPT1A (ab83862, abcam, Cambridge, MA, USA), at 4 °C overnight. After washing with PBS, the cells were incubated with secondary antibody (SA00013-4, proteintech, Wuhan, China) for 2 h at room temperature. The cell nuclei were then colored with 4′,6′-diamidino-2-phenylindole (DAPI), and the fluorescence images were analyzed using a fluorescence microscope (Zeiss, Tokyo, Japan). Changes in protein levels of CPT1A after overexpression and knockdown of CPT1A detected using immunofluorescence are shown in the Supplementary Materials (Supplementary Materials Figures S7 and S8).

### 4.14. Western Blot Analysis

Western blotting was carried out using an SDS-PAGE Electrophoresis System. Adherent cells extracts were prepared and transferred to PVDF membranes. The primary antibodies for this experiment were as follows: anti-β-actin (1:6000, BM0627, BOSTER, Wuhan, China), anti-p-p38-MAPK (1:1000, 3285S, Cell Signaling Technology, Danvers, MA, USA) and anti-p38-MAPK (1:1000, 4, Cell Signaling Technology, Danvers, MA, USA). Target proteins were visualized by the enhanced chemiluminescence (ECL) detection systems (Thermo, Waltham, MA, USA).

### 4.15. Statistical Analysis

All the data were provided as “means ± SEM”. Analysis of variance was performed to compare significance with GraphPad prism 8.0 software, followed by Duncan’s multiple comparisons test. When “*” *p* < 0.05, “**”*p* < 0.01, “***”*p* < 0.0001, the differences were considered significant.

## 5. Conclusions

**Figure 7:**
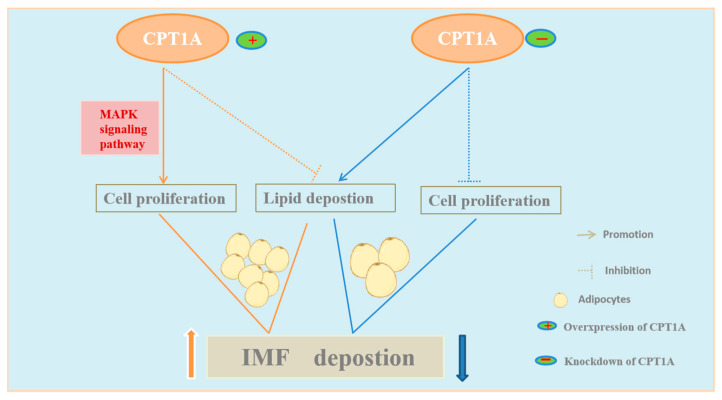
Schematic diagram of the regulation of IMF deposition by *CPT1A*. Significant enrichment of MAPK signaling pathway was found by RNA-seq sequencing and analysis of CPT1A knockdown and controls. Overexpression of *CPT1A* inhibits lipid deposition, but promotes cell proliferation via the MAPK signaling pathway, thereby altering the distribution of lipids in cells to more cells with fewer lipid droplets, ultimately allowing for increased IMF deposition. Knockdown of *CPT1A* inhibits cell proliferation, but promotes lipid deposition, thereby altering the distribution of lipids in cells to fewer cells with more lipid droplets, ultimately allowing for decreased IMF deposition.

Our current work demonstrates that the expression variation of *CPT1A* may cause the reconstruction of lipid distribution between cellular triglyceride deposition and cell proliferation in goat intramuscular precursor adipocyte. Furthermore, we demonstrate that CPT1A promotes the proliferation of goat adipocytes through the MAPK signaling pathway (Figure 7). This work widened the genetic regulator networks of IMF formation and delivered theoretical support for improving meat quality from the aspect of IMF deposition.

## Data Availability

Original data can be made available upon request. The names of the repository/repositories and accession number(s) can be found below: NCBI Sequence Read Archive and Bio project number PRJNA979117.

## Supplementary Materials

The following supporting information can be downloaded at: https://www.mdpi.com/article/10.3390/ijms241713415/s1.
