# Supplementary material for: Expression Variation of CPT1A Induces Lipid Reconstruction in Goat Intramuscular Precursor Adipocytes
Source: Int J Mol Sci. 2023 Aug 29;24(17):13415. doi: 10.3390/ijms241713415 (PMC10488119; doi:10.3390/ijms241713415)
Supplement: Supplementary file 1 [file ijms-24-13415-s001.zip › Supplementary Figures.pdf]

Supplementary Materials

Expression Variation of *CPT1A* Induces Lipid Reconstruction in Goat Intramuscular Precursor Adipocytes

Yinmei Tang [1], Wenyang Zhang [2], Yinggui Wang [1], Haiyang Li [1], Changhui Zhang [1], Yong Wang [1,2], Yaqiu Lin [1,2], Hengbo Shi [3], Hua Xiang [1,2], Lian Huang [1] and JiangJiang Zhu [1,2,*]

* Correspondence: zhujiang4656@hotmail.com

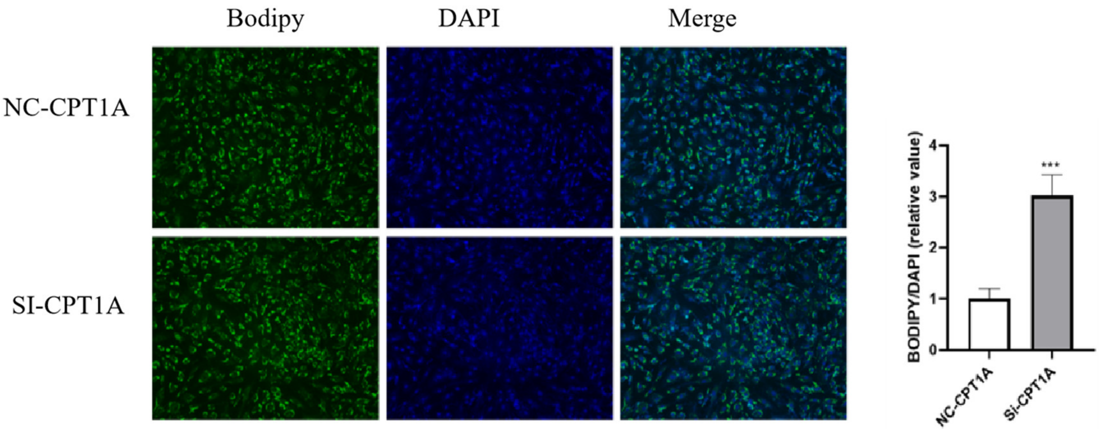

Figure S1. Knockdown of CPT1A after Bodipy staining, quantification of lipid droplet content. Data was presented as mean ± SEM for three independent experiments. **P<0.05*, ***P<0.01*, ****P<0.000*

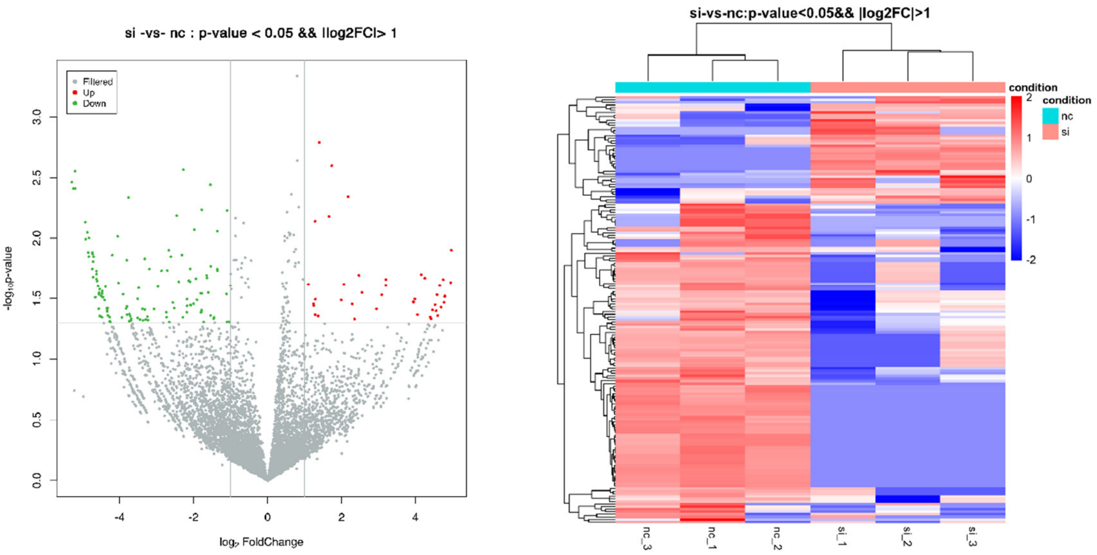

Figure S2. Volcanic map of DEGs (left), clustering map of DEGs (right).

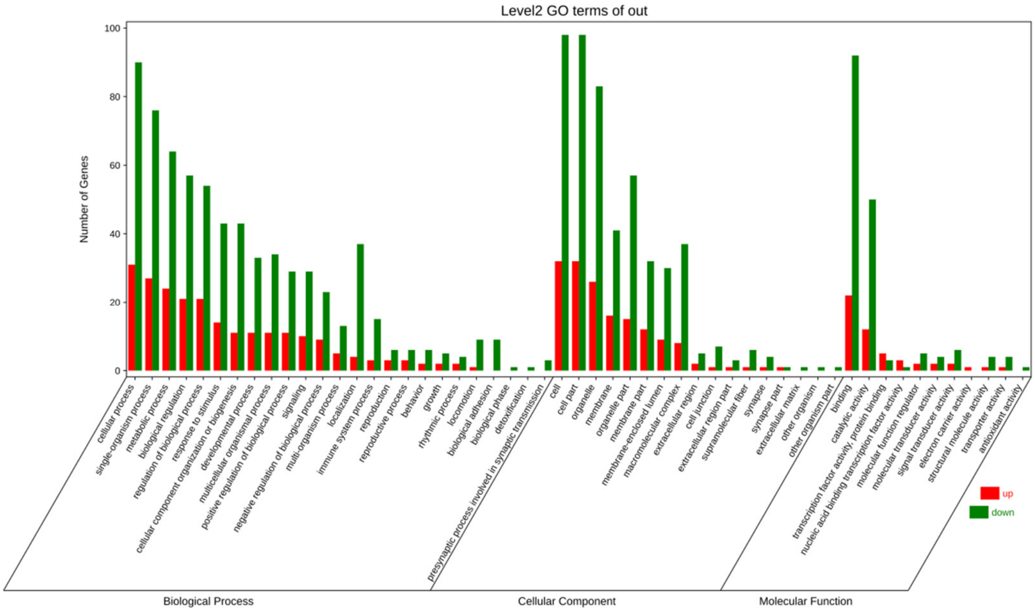

Figure S3.GO enrichment analysis of DEGs. The GO terms of up-regulated and down-regulated DEGs was differentiated by red and green color, respectively.

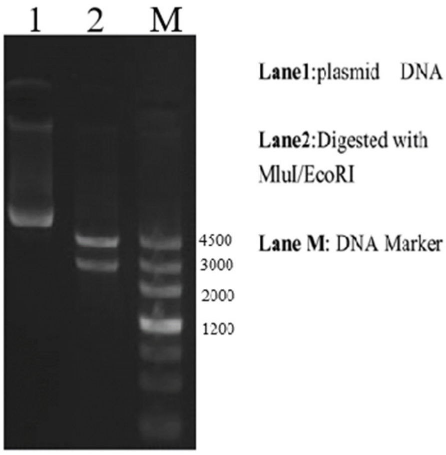

Figure S4. Overexpression vector construction. Gel electrophoresis of double digest products.

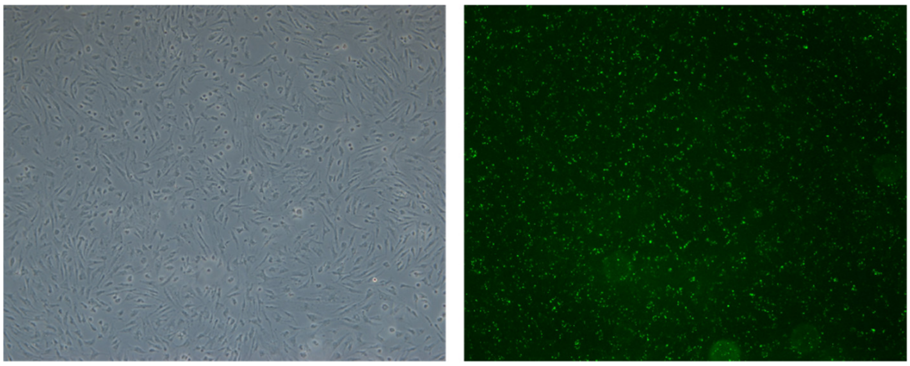

Figure S5. Validation of transfection systems. The images shows intramuscular precursor adipocytes transfected with fluorescently labelled (FAM) si-RNA, photographed in natural light on the left and fluorescently on the right.

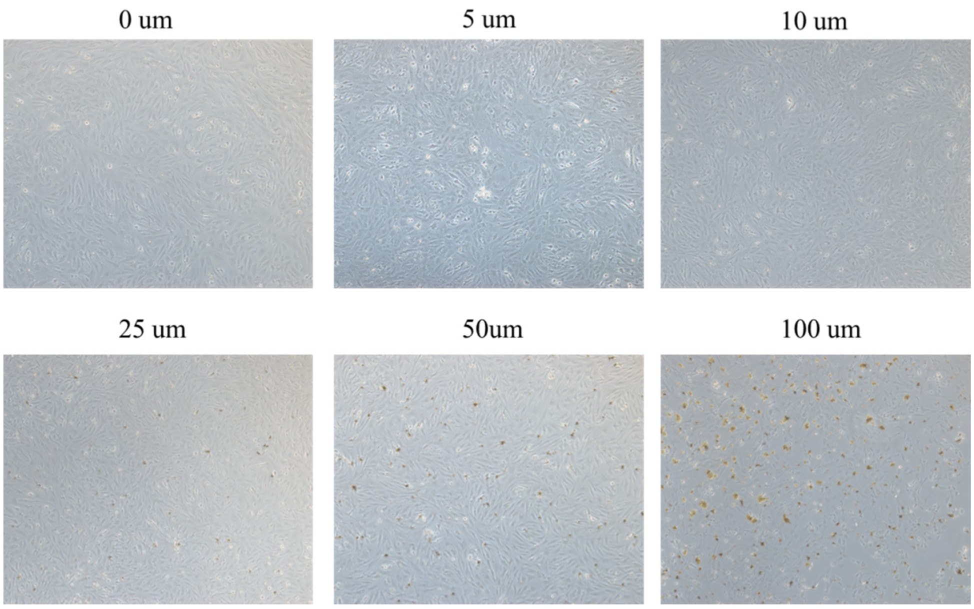

Figure S6. MAPK inhibitor (PD169316) concentration screening. The effect of MAPK inhibitor (PD169316) on intramuscular adipocytes growth at 24 h after PD169316 (5, 10, 25, 50 and 100 uM) or DMSO treatment. N=3.

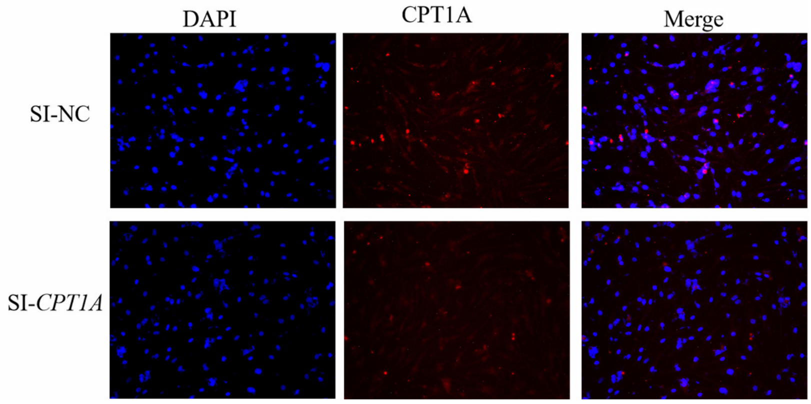

Figure S7. Knockdown of *CPT1A* and negative control immunofluorescence assay. Antibody Dilution : 1：300. Anti-CPT1A antibody (ab83862) comes from abcam. Sencondary Antibody:GoraLite 594-conjugated Goat Anti-Rabbit IgG (SA0013-4) comes from proteintech.

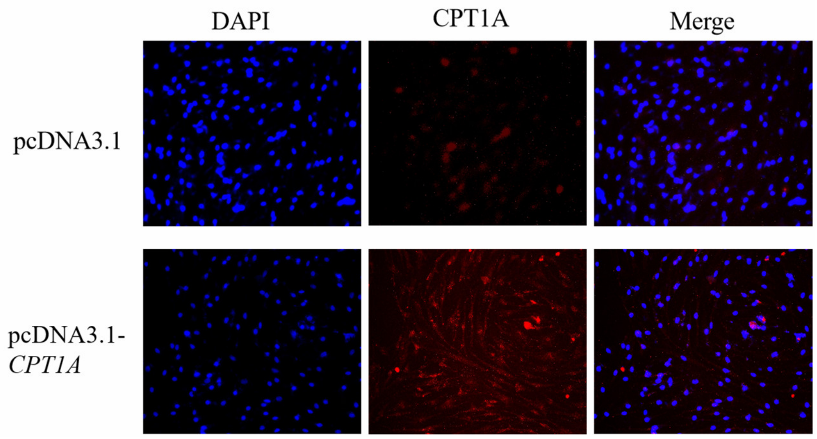

Figure S8. Overexpression of CPT1A and pcDNA3.1 immunofluorescence assay. Antibody Dilution : 1：300. Anti-CPT1A antibody (ab83862) comes from abcam. Sencondary Antibody:GoraLite 594-conjugated Goat Anti-Rabbit IgG (SA0013-4) comes from proteintech.
